# *In Situ* Surface-Enhanced Raman Spectroscopy on Organic Mixed Ionic-Electronic Conductors: Tracking Dynamic Doping in Light-Emitting Electrochemical Cells

**DOI:** 10.1021/acsami.4c00684

**Published:** 2024-05-23

**Authors:** Mohammad Javad Jafari, Jonas Oshaug Pedersen, Samira Barhemat, Thomas Ederth

**Affiliations:** † Division of Biophysics and Bioengineering, IFM, Linköping University, Linköping 581 83, Sweden; ‡ Department of Vision Inspection, Mabema AB, Linköping 584 22, Sweden

**Keywords:** light-emitting electrochemical cell, surface-enhanced Raman spectroscopy, electrochemical doping, time-resolved, MEH-PPV

## Abstract

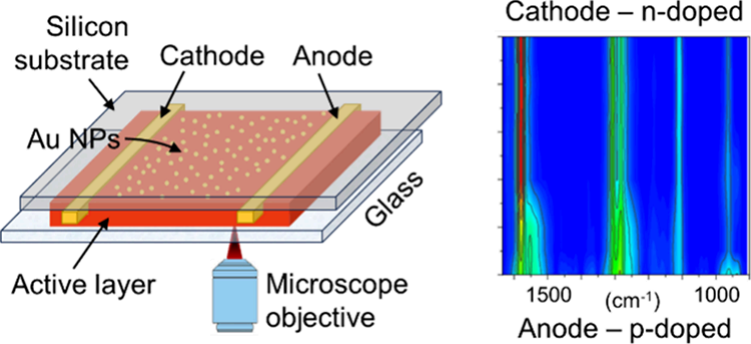

In the domain of organic mixed ionic–electronic conductors (OMIECs), simultaneous transport and coupling of ionic and electronic charges are crucial for the function of electrochemical devices in organic electronics. Understanding conduction mechanisms and chemical reactions in operational devices is pivotal for performance enhancement and is necessary for the informed and systematic development of more promising materials. Surface-enhanced Raman spectroscopy (SERS) is a potent tool for monitoring electrochemical evolution and dynamic doping in operational devices, offering enhanced sensitivity to subtle spectral changes. We demonstrate the utility of SERS for *in situ* tracking of doping in OMIECs in an organic light-emitting electrochemical cell (LEC) containing a conjugated polymer (poly[2-methoxy-5-(2-ethylhexyloxy)-1,4-phenylenevinylene]; MEH-PPV), a molecular anion (lithium triflate), and an electrolyte network (poly(ethylene oxide); PEO). SERS enhancement is achieved via an interleaved layer of gold particles formed by spontaneous breakup of a deposited thin gold film. The results successfully highlight the ability of SERS to unveil time-resolved MEH-PPV doping and polaron formation, elucidating the effects of triflate ion transfer in the operating device and validating the electrochemical doping model in LECs.

## 1 Introduction

Organic mixed ionic–electronic conductors (OMIECs) are versatile materials that have gained importance due to their ability to facilitate the transport of both ions and electronic charges.^1−5^ These soft, often polymer-based conductors have evolved alongside the broader development of organic π-conjugated polymers and small molecules.^1,4^ OMIECs are typically polymeric and can be categorized based on their ion- and electron-conducting components.^4^ OMIEC materials offer mixed conductivity and chemical adaptability, making them suitable for application in diverse fields, such as batteries,^6^ supercapacitors,^7^ light-emitting electrochemical cells,^8−11^ and organic electrochemical transistors for various sensing and neuromorphic computing applications.^12^ Underlying these applications is the dynamic nature of the OMIECs during operation, influenced by factors such as time, voltage, chemical composition, operation temperature, and environment. Operating the OMIEC devices results in notable alterations in composition, structure, and component density. These changes occur due to the redistribution of ions and electrons within the active material. In OMIECs where ions are chemically bonded to conjugated components, the electric field causes charge redistribution within the material.^13^ In the presence of an ionically conducting solid polymeric electrolyte, these ions become free entities during device operation, establishing dynamic equilibria and inducing mass transport at the interface.^14^

Light-emitting electrochemical cells (LECs), as a subset of OMIECs, exhibit the unique ability to generate light through reversible electrochemical reactions.^11^ In LECs, the active materials are heterogeneous blends or complex systems of electrically conductive conjugated polymers and solid polymeric electrolytes, where mobile ion carriers become free species during device operation. The diffusion of these ions is essential for various aspects of LEC performance, including device turn-on time and polymer doping.^1,14,15^ Ion redistribution within LEC devices is influenced by factors such as ionic conductivity,^16,17^ active material thickness,^18^ applied bias, and operating temperature.^19,20^ These variables contribute to turn-on times, which span from milliseconds to hours, representing the duration required for the p- and n-doped regions to establish a p–n junction.^14,21,22^ Two models, the electrodynamic model (ED) and the electrochemical doping model (ECD), have been proposed to explain the conduction mechanism in LECs when an external bias is applied.^14,23−25^ In the ED model, the applied potential primarily drops over the electric double layers (EDLs) near the electrode interfaces, resulting in a weak electric field within the bulk polymer and dividing the active layer into three regions. In contrast, the ECD model suggests that the electric field drops over the EDLs only as much as needed to create ohmic contacts, establishing an efficient electric field that facilitates increased charge carrier injection into the active layer and leads to the oxidation/reduction of the conjugated polymer. As charge carriers accumulate, ions move toward electrodes with opposite charges, eventually leading to complete ion separation and the formation of an electrical junction, resulting in a steady-state device. Additionally, the polymer forms ohmic contacts, creating highly conductive p- and n-doped regions at the electrodes, with the remaining potential difference dropping at a narrow p–n junction region.^14,23,24^

A deeper understanding of processes like doping, ion migration, and electronic and chemical reactions within OMIECs can provide valuable insights into the function of these devices and pave the way for future advancements in solid electrochemistry technology. Numerous studies have been conducted to elucidate the operational mechanisms in electrochemical systems. Surface-sensitive techniques such as scanning Kelvin probe microscopy (SKPM),^25^ electric force microscopy (EFM),^26^ atomic force microscopy (AFM),^27^ and scanning tunnelling microscopy (STM),^28^ can yield real-time morphological insights into electrode surfaces and dynamically characterize potential profiles. Mass spectroscopic techniques have been used to investigate spatial ion density distributions and to detect electrochemically generated intermediates and products.^15,29^ X-ray techniques, such as X-ray diffraction (XRD),^30^ synchrotron X-ray scattering,^5,31^ X-ray tomography,^32^ and X-ray absorption spectroscopy (XAS)^33^ are utilized for operando studies of electrochemical processes in batteries, offering valuable insights into both structural and surface electronic properties.^34^ In general, time-resolved studies under relevant device conditions are crucial for capturing dynamic changes and establishing meaningful structure–property relationships,^2,4,35−37^ and systematic design and optimization of OMIEC devices depend on use and development of operando characterization techniques with sufficient sensitivity.^2,4^

Vibrational and optical spectroscopic methods are extensively used as spectroelectrochemical techniques to investigate material structures, monitor time-resolved *in situ* or operando chemical reactions,^35,37−39^ perform chemical imaging and material mapping,^2,36,40,41^ assess local species density,^35^ and explore electrode–electrolyte interfacial interactions^36−39^ during electrochemical processes. Raman spectroscopy stands out as a widely used nondestructive spectroelectrochemical tool for tracking doping mechanisms and studying electrochemical processes in solid-state electrochemical devices. It has the capability to perform structural analyses ranging from the bulk electrolyte to the diffusion layer within the EDL, all the way to surface-adsorbed molecules and electrode materials.^34,36,42,43^ Surface-enhanced Raman spectroscopy (SERS) leverages electric field enhancement via surface plasmon resonance to detect subtle signals from molecules at both the electrode surface and the electrode–electrolyte interface in electrochemical systems and excels at capturing weak signals in these environments.^43^ The electric field enhancement is the strongest at “hot spots” formed between closely spaced particles or at sharp features of individual particles, and analytes located at these features account for a large part of the total signal.^44^ The increased sensitivity provided by SERS makes this a preferred method for time-resolved studies of OMIECs aimed at improving our understanding of dynamic changes under operation.

**Figure 1:**
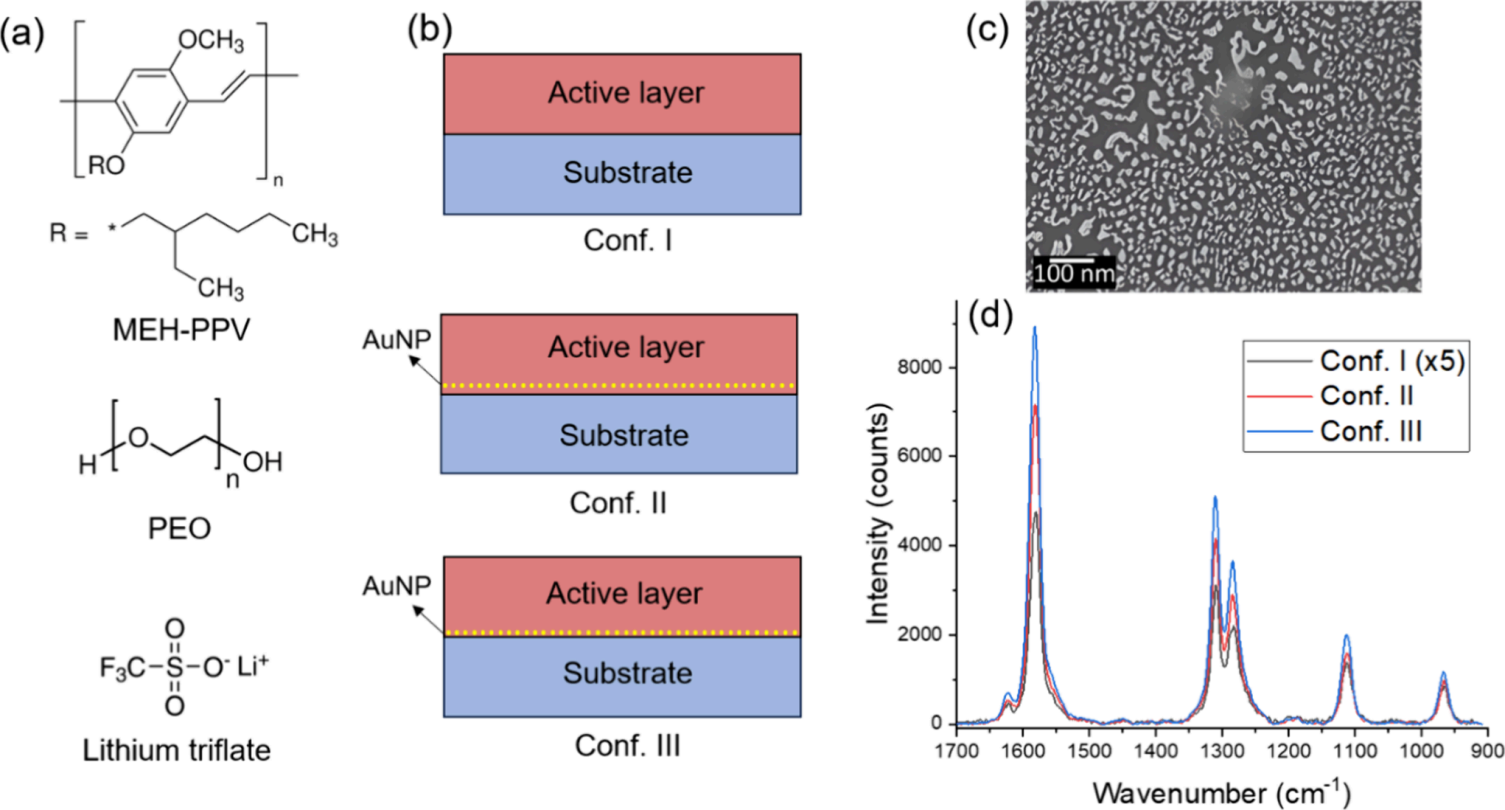
(a) Molecular structure of the active material components and (b) three different configurations of active layer on Si substrates, with different locations of the layer of gold nanoparticles (AuNPs). (c) SEM image of a 40 Å Au layer on top of a Si substrate showing disjunct particles formed by dewetting of the gold from the substrate and (d) Raman spectra of the active material in the various configurations (λ_0_ = 785 nm, 5 mW, 30 s). Note that the spectrum for configuration I has been scaled ×5 to facilitate comparison of the shapes between the three spectra.

Previously, we demonstrated the utility of FTIR microscopy for tracking and mapping ion mobility and polymer doping within the active layer of planar LECs as an OMIEC system.^35^ In this article, we have used Raman spectroscopy, enhanced by gold nanoparticles formed via spontaneous breakup of thermally evaporated gold films on the substrate of planar LEC devices, to create a sensitive SERS substrate, with a view to improving the understanding of dynamic processes leading to the formation of the light-emitting junction. Earlier studies reported that a uniform and dense distribution of light-emitting regions in planar LEC devices can be obtained by including conducting particles into the LEC films prepared by spin-coating, or from a layer of metallic nanoislands formed onto the LEC film. These micro- or nanoislands play a crucial role in the formation of smaller light-emitting domains within thin film LECs.^45,46^ Here, a procedure akin to the latter method was used but using the additional particles for SERS enhancement instead. The increased sensitivity obtained with these substrates allows for time-resolved *in situ* tracking of polymer doping profiles through ion migration in LECs during device operation under applied bias, establishing SERS as a very useful method for studying OMIECs. The active layer in our experiments consists of poly[2-methoxy-5-(2-ethylhexyloxy)-1,4-phenylenevinylene] (MEH-PPV) as the light-emitting polymer and poly(ethylene oxide) (PEO) as a solid electrolyte doped with lithium triflate (LiCF_3_SO_3_) salt (see Figure 1a). To achieve high SERS sensitivity, various configurations of the active material layer were prepared, and the most sensitive configuration was selected for measurements under applied bias. Our experimental setup focuses on a planar LEC with a 2 mm interelectrode gap. We continuously capture Raman spectra from the region near the anode electrode to perform spectroelectrochemical characterization of the operating device at room temperature. This allows us to track the p-doping profile and polaron formation dynamics *in situ* under bias using SERS.

## 2 Methods

### Materials

To prepare the active material, poly[2-methoxy-5-(2-ethylhexyloxy)-1,4-phenylenevinylene] (MEH-PPV, *M*_n_ = 40 000–70 000 Da, Sigma-Aldrich) was dissolved in chloroform at a concentration of 10 mg/mL. Poly(ethylene oxide) (PEO, *M*_v_ = 6 × 10^5^, Sigma-Aldrich) and lithium triflate (LiCF_3_SO_3_, Sigma-Aldrich) were dissolved in cyclohexanone to prepare a solution with concentrations of 13.5 and 2.5 mg/mL, respectively (see also Figure 1a). This procedure closely follows the method described by Edman and co-workers,^10^ and stock solutions were stored in a refrigerator.

### Active Layer Preparation

To investigate the effect of a thin gold layer on Raman spectral enhancement, we tested three different configurations for the active material film on top of a Si substrate, as illustrated in Figure 1b. The active material was prepared by diluting the stock solutions by a factor of 5 and then mixing the two solutions in a 1:1 ratio. This resulted in the active material solution with a mass ratio of MEH-PPV:PEO:LiCF_3_SO_3_ at 1:1.35:0.25. Preliminary studies showed that spin-coating the active layer onto gold particle layers resulted in uneven distributions of the particles, which could negate the surface enhancement effect. Hence, we used drop-casting to apply the active layer onto any deposited particle layers. In configuration I, the active material was drop-casted onto the Si substrate and dried. In configuration II, the active material was spin-coated onto the substrate at 1000 rpm for 30 s, resulting in an approximately 100 nm-thick dried film. Following this, a 40 Å-thick gold layer was thermally evaporated onto the active layer. Then, a second layer of the active material was drop-casted over the gold layer and dried. In configuration III, we began by thermally evaporating a 40 Å-thick gold layer directly onto the Si substrate. Subsequently, the active material was drop-casted onto the gold-coated Si substrate and dried, using 20 μL of active material solution per cm^2^, resulting in dried films with estimated thicknesses of 1–2 μm.

### Device Fabrication

**Figure 2:**
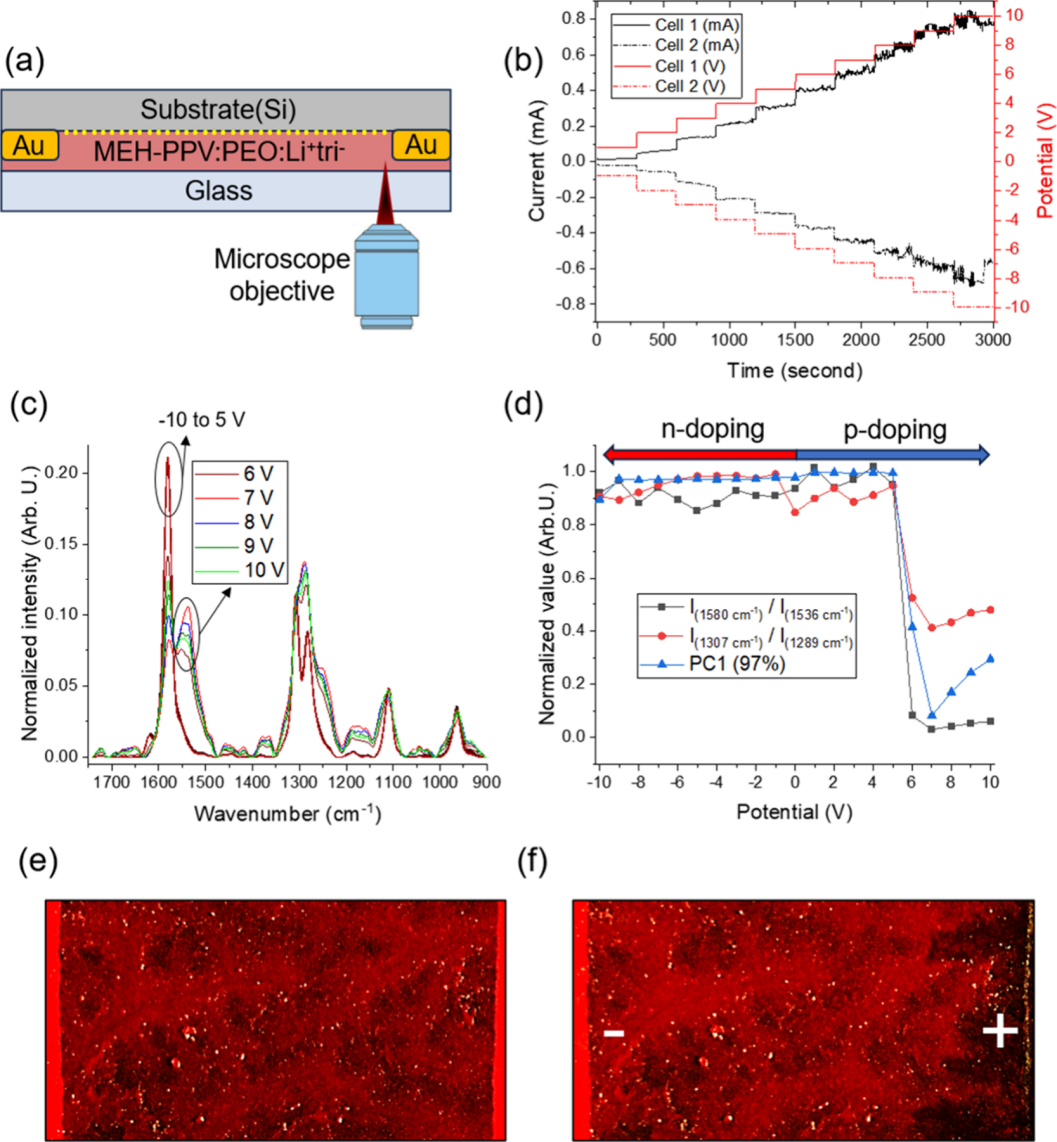
(a) Cell schematic for Raman spectroscopy and (b) current and potential versus time for both cells under applied bias. (c) Normalized Raman spectra of the active material near the working electrode under each potential step (λ_0_ = 785 nm, 5 mW, 300 s) with (d) potential dependence of band intensity ratios and PC1 evolution due to MEH-PPV doping. Images of the planar LEC device (e) before applying bias and (f) after applying a 10 V bias for 400 s at room temperature. The electrodes are visible at the left and right image edges, but the anode is hidden under the doped polymer in (f).

After comparing the Raman spectra of various configurations (as discussed in the results and discussion section), we selected configuration III for the preparation of the LEC devices. We prepared electrodes by depositing Ti and Au onto a Si substrate. The Ti adhesion layer had a thickness of approximately 40 Å, while the Au electrodes were deposited to a thickness of 2000 Å. The electrodes are separated by a 2 mm gap and are 5 mm wide; see Figure 2a. The wide electrode gap was intentionally prepared to facilitate extended ion transfer within the devices, making it easier to dynamically track doping profiles. The 40 Å-thick Au layer forming the particles was deposited after preparing the electrodes. The subsequent preparation steps involving the active material were carried out inside a glovebox under a nitrogen atmosphere, as described above, and dried at 120 °C for 30 min after drop-casting. The active material layer between the electrodes was encapsulated to prevent ambient degradation of the active layer^8^ by covering the devices with a coverslip taped to the supporting Si substrate at the edges. Nine samples were prepared in configuration III, and the consistency between them was checked with resistance measurements, resulting in a variation of ±13%.

### Raman Spectroscopy

The spectroelectrochemical Raman and SERS investigations were carried out on a Nikon Ti-E inverted microscope using a long working distance 60× air objective (Nikon CFI S Plan Fluor, 0.7 N.A). A precision sample stage (Nikon TI-S-ER, repeatability <0.5 μm) allows accurate positioning of the Raman illumination at known distances from the electrodes. We intended to measure as close to the anode as possible, but at the same time, to avoid excitation laser reflections from the electrode. The shortest distance from the electrode edges where these were eliminated was ca. 10 μm. Laser excitation lines at 532 and 785 nm were used with output powers of 0.05 and 5–10 mW, respectively, and illuminating the sample via a rear port on the microscope. Scattered light was extracted from the same light path via a beamsplitter and led to an Andor Kymera 328i Raman spectrograph via an optical fiber. The spectrograph uses a diffraction grating of 600 l/mm (blazed at 500 nm) and is fitted with a thermoelectrically cooled (−80 °C) EMCCD camera (Andor Newton DU970P-BVF). All spectra were collected using accumulate or time series modes, depending on the application. Afterward, the spectra were processed as described below.

### Spectral Processing

The spectral range of 1700–900 cm^–1^ was selected for data analysis. The spectra obtained through the kinetic measurement method were subjected to smoothing using a Savitzky–Golay filter (window size = 13, poly order = 3). Subsequently, all spectra were baseline-corrected using the asymmetric least-squares (ALS) algorithm (iterations = 10, windows size = 200, asymmetry parameter = 0.001). To facilitate the comparison of spectra with varying doping profiles, we applied vector normalization across the entire spectral range.

Principal component analysis (PCA) is a technique used to reduce the dimensionality of data by transforming it into a set of orthogonal eigenvectors, which are linear combinations of the original variables. These eigenvectors indicate the directions of maximum variance in the data while preserving information about the data point variations.^47^ This transformed space is defined by the principal components (PCs), and most of the variance in the original data is captured by the first few PCs, typically one, two, or three. PCA was applied to condense the extensive array of correlated wavenumbers into new compact data sets. This procedure was independently executed for selected spectral regions to examine how the doping profile of MEH-PPV influenced the spectral changes. For the subsequent data analysis, only the first principal component (PC1) was used.

## 3 Results and Discussion

To obtain the most significant spectral enhancement in Raman spectra, three different configurations of active materials on a Si substrate were prepared, as represented in Figure 1b. In these, the formation of gold nanoparticles for Raman enhancement relies on spontaneous island formation caused by poor wetting of the gold film to the substrate, whether it is the silica or the active layer, and subsequent island formation via capillary instabilities.^48^*I*–*V* curves acquired between the electrodes for voltages from 0 to +10 V after this deposition step resulted in currents in the nA range and with no clear correlation with applied voltage, indicating that there is no conductive path formed between the electrodes by this additional gold layer. Devices prepared with and without the gold-particle-forming layer also showed no significant differences in resistance between the electrodes. This is consistent with literature suggesting that dilute layers of metal nanoparticles have little influence on the performance of electronic devices.^49^ A scanning electron microscopy (SEM) image of the Au film on the Si substrate is shown in Figure 1c. Notably, this Au film is composed of nanometer-sized Au particles with different sizes. The SEM image demonstrates the characteristic morphology of a thin metallic layer on a Si substrate, filled with numerous narrow gaps interspersed between the particles. These gaps are potential hot spots where Raman spectra can be significantly amplified.^50,51^ We observed a strong fluorescence background using a 532 nm excitation laser (Figure S1 in the Supporting Information). To tackle this issue, we switched to using a 785 nm excitation laser in our study. As shown in Figure 1d, the intensities of the Raman spectra for configurations II and III are amplified, with enhancements of approximately 7 and 10 times when compared to configuration I (without the Au layer), respectively. Note that the spectrum for configuration I is scaled ×5 in Figure 1d. Based on the geometries shown in Figure 1b, configuration II could be expected to yield a more intense signal since the particles are surrounded by the active layer material but facing only a half-space of material in configuration III. The opposite result in Figure 1d might be attributed to redissolution of the bottom spin-coated layer during drop casting, which may impact the distribution of gold nanoparticles, thereby reducing the SERS enhancement. Additionally, the results indicate that the shape and position of the peaks remain nearly identical across the three spectra, suggesting the absence of any chemical reactions between the Au layer and the active material layer. Furthermore, this also allows us to do normal band assignments since we do not need to consider interaction-based spectral differences caused by chemical enhancement. Based on these results, we selected configuration III, with the Au film deposited directly onto the Si substrate due to its simpler preparation process, for all subsequent measurements in this study.

The modest enhancement of the total signal in Figure 1d is consistent with previous studies of SERS in bulk materials.^52^ The largest difference in signal intensity between SERS and regular Raman occurs for (sub)monolayers and thin films, where the surface sensitivity of SERS allows detection of very weak signals from minute amounts of material. When particles are encapsulated in a bulk material, only a small fraction of the material is available for plasmonic enhancement, while the vast majority of analyte is too far away to benefit from the enhancement. The enhancement factor (EF) can be estimated to the order of ∼50 in this case (see Supporting Information, p. S8). This estimate relies on some simplifying assumptions, but suffices to give a (conservative) estimate of the enhancement. The calculated value is in line with those from numerical simulations of monolayers of gold NPs without sharp features at approximately 40–50% surface coverage.^53^ Additionally, the 785 nm laser wavelength is far from the plasmon peak of the nanoparticles, which is closer to 610 nm (Figure S2). The modest EF should also be considered from the perspective of the very simple procedure for preparation of the particle layer; high EFs are usually associated with elaborate procedures for preparing and/or distributing nanoparticles, which are avoided here.

By a comparison of the Raman spectra of the active material and MEH-PPV (Figure S3a), it is evident that these two spectra closely resemble each other. This similarity indicates our ability to track the doping profile of MEH-PPV within the LEC device. The strong band at 1580 cm^–1^ corresponds to the symmetric CC stretching vibration of the phenyl ring,^54−57^ while two adjacent weaker bands at 1621 and 1554 cm^–1^ are attributed to the asymmetric CC stretching of vinylene and the CC stretching on the phenyl, respectively.^54,58,59^ The two bands observed at 1307 and 1283 cm^–1^ are associated with the asymmetric CC stretching and CC interring stretching coupled with CC–H bending of the phenyl ring, respectively.^54,56,57,60^ It is worth noting that a weaker peak linked to vinylene CC–H bending might be expected around 1330 cm^–1^, but it could be overshadowed by a neighboring strong band.^61,62^ The results also reveal a weak peak around 1180 cm^–1^, attributed to CC–H ring bending, and a band at 1108 cm^–1^, which emerges from the mixing of C–C stretching and C–H in-plane-bending vibrations within the phenyl group.^55−58^ The band at 963 cm^–1^ is attributed to the out-of-plane CH bending of the vinylene group. This particular vibration arises from the dihedral angle between two monomer units, which is forbidden in the Raman spectrum of planar polymer configuration.^54,57^

As previously explained, the LEC devices were prepared to monitor the dynamic doping of MEH-PPV, as illustrated in Figure 2a. To obtain Raman spectra under positive and negative bias, we used two different LEC cells in which we applied step potentials ranging from 0 to +10 (cell 1) or from 0 to −10 V (cell 2). Each step was maintained for a duration of 300 s, as shown in Figure 2b. The rationale for using two different cells lies in the fact that once potential is applied to the devices, the junction becomes fixed, making it impossible to alter the junction polarization without replacing the entire cell. To track the doping profile of MEH-PPV at each potential, Raman spectra were collected near (∼10 μm) the working electrode. All measurements were done at room temperature, and average spectra were obtained in each step. Under a positive bias (cell 1), the working electrode functions as the anode, while under negative polarization (cell 2), the working electrode serves as the cathode. As previously demonstrated using FTIR spectroscopy,^35^ this configuration enables us to track p-doping near the electrode under positive bias and n-doping under negative bias. By using Raman spectroscopy, we expect to observe polaron formation under applied positive bias due to oxidation, while under negative bias, we do not anticipate the presence of any polarons.

Figure 2c presents the *in situ* normalized Raman spectra of the active material near the working electrode under various applied biases. The findings indicate that there is no significant alteration in the spectra under applied negative bias, which corresponds to n-doping. Similarly, there are no significant spectral changes for applied positive biases up to 5 V. At low potentials, which are too small to overcome the band gap of MEH-PPV (approximately 2.3 eV), double layers form near the electrodes. By an increase in the applied potential, the formation of the junction begins, which can be a slow process. When electrochemical equilibrium is established, mobile ions will redistribute. The onset of electrochemical doping can also be delayed due to the existence of an overpotential.^63^

However, a spectral evolution can be observed under positive bias exceeding 5 V due to MEH-PPV p-doping (each spectrum is shown individually in Figure S4). Due to the wide interelectrode gap in our devices, which was chosen to facilitate slow ion migration for tracking doping evolution via Raman spectroscopy, no light emission appears in our devices at these potentials. However, at higher voltages, such as 200 V, light emission appears from a region near the anode (Figure S5). At these potentials, there is also a small contribution to the luminescence from the included gold particles, as discussed in the Supporting Information in relation to Figure S5. By comparing the images of the devices before (Figure 2e) and during (Figure 2f) the application of a 10 V bias, we observed a visible color change in the active layer, which is related to MEH-PPV doping. This doping emerges first at the anode and visibly progresses into the interelectrode area with time, demonstrating a continuous progression of the doping.

**Figure 3:**
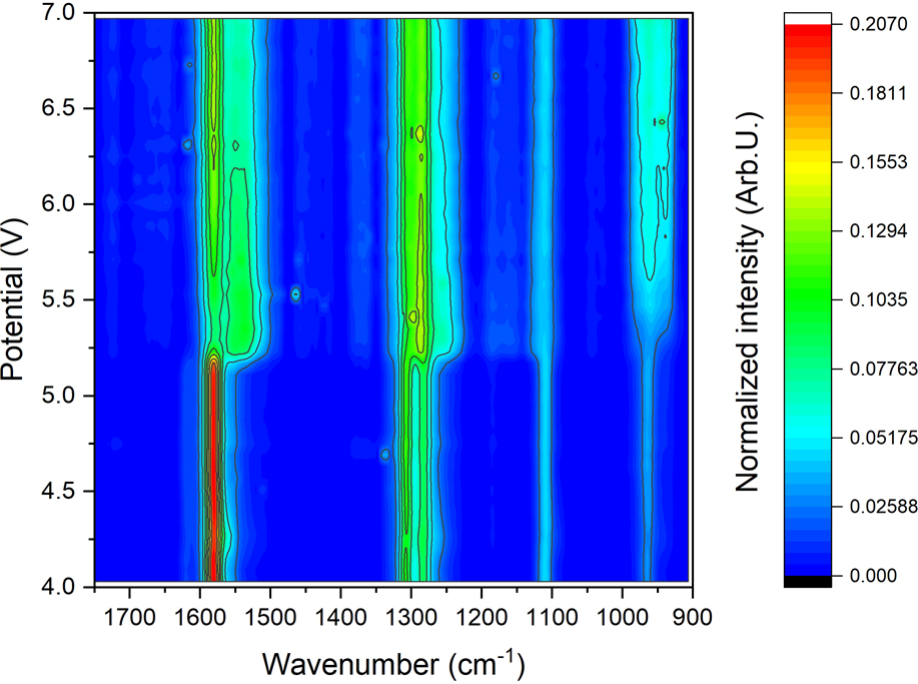
Raman spectra map of the active material versus applied bias. MEH-PPV doping is initiated at around 5.2 V (λ_0_ = 785 nm, 5 mW, 60 s).

The spectral evolution at biases over 5 V reveals a decrease in the intensity of the peak at 1580 cm^–1^, associated with the CC stretching vibration of the phenyl ring in neutral MEH-PPV. Simultaneously, a new peak emerges at approximately 1536 cm^–1^. This new band is attributed to the CC stretching vibration of the doped segment,^61,62^ due to structural and electronic modifications in the MEH-PPV backbone, as well as ring conversion during polymer doping (see Figure S6 for the structural changes to MEH-PPV induced by p-doping, and a graphical illustration of how these relate to the spectral changes). They are also consistent with polaron injection and the formation of a quinoid structure upon polymer oxidation (p-doping).^35^ Accompanying the appearance of the new band, a shoulder is observed around 1546 cm^–1^. This shoulder corresponds to the CC stretching of vinylene in doped MEH-PPV, and it is notable that the band at 1621 cm^–1^, associated with the CC stretching of vinylene in neutral MEH-PPV, diminishes as a result.^61,64^ Vibrational modes that are coupled to the π-electron system exhibit remarkable sensitivity to these modifications,^61^ which can indicate a partial (incomplete) conversion of undoped MEH-PPV from a benzoid structure to a quinoid structure during the p-doping process (see Figure S6). The bands at 1307 and 1283 cm^–1^, which correspond to phenyl CC interring stretch and CC–H bending in neutral MEH-PPV, exhibit downshifts to 1288 and 1258 cm^–1^, respectively, upon doping.^61,64,65^ The peak assignments for both the neutral and doped active materials are summarized in Table 1. Electrochemical doping results in significant differences between the ordered and disordered domains within conducting polymer films, as reported in previous studies.^66^ These differences can account for the variations observed in the intensity, shape, and position of various CH vibrations in doped MEH-PPV. Specifically, vibrations associated with the vinylene out-of-plane CH bending appeared as an overlapped band, with two peaks at 975 and 955 cm^–1^ within the doped segment. This change can be attributed to the reordering of the MEH-PPV configuration induced by doping, leading to changes in the dihedral angle between two monomer units. To monitor the doping profile more accurately, we employed a sweeping bias ranging from 4 to 7 V at a scan rate of 1 mV/s (Figure S7), all while continuously tracking the MEH-PPV doping profile by recording *in situ* Raman spectra near the anode at room temperature. In Figure 3, Raman spectra are plotted for these applied biases. The results indicate that MEH-PPV doping initiates at around 5.2 V. It is worth noting that the onset potential for electrochemical doping can be influenced by various factors, including temperature and the width of the interelectrode gap, as ion mobility and diffusivity are temperature-dependent, defined by the Einstein relation.^1^

**Table 1:** Band Assignments for Neutral and p-Doped Active Material (MEH-PPV)

neutral MEH-PPV		doped MEH-PPV	
band position (cm^–1^)	assignment	band position (cm^–1^)	assignment
1621	vinylene CC stretch^54,55,58−60^	1580	phenyl CC stretch^54−60^
1580	phenyl CC stretch^54−60^	1546	vinylene CC stretch^61,64^
1554	phenyl CC stretch^54,57,59,60^	1536	phenyl CC stretch^54,61,62^
1307	phenyl CC stretch + phenyl CC-H bending^58,60^	1336	vinylene CC-H bending^61,62^
1283	phenyl CC interring + phenyl CC-H bending^56−58^	1288	phenyl CC stretch + phenyl CC–H bending^61,64,65^
1180	phenyl CC–H bending	1258	phenyl CC interring + Phenyl CC–H bending^61,64,65^
1108	phenyl C–C stretching + C–H in-plane-bending^55,56,58^	1185, 1160	phenyl CC–H bending^61^
963	vinylene out-of-plane CH bending^54,56,58^	1125	phenyl C–C stretching + C–H in-plane-bending^61^
		975, 955	vinylene out-of-plane CH bending^54,64^

To illustrate the structural evolution of MEH-PPV induced by doping, we track this transformation by monitoring the shift in the position of the phenyl group Raman bands. Ratios of the intensities of these peaks, measured and normalized, are depicted in Figure 2d, showing *I*_1580 cm^–1^_/ *I*_1536 cm^–1^_ and *I*_1307 cm^–1^_/ *I*_1289 cm^–1^_ over the range of investigated biases. Additionally, we used PCA to analyze the normalized Raman spectra. The first principal component (PC1) shows the differences between neutral, n-doped, and p-doped MEH-PPV. The PC1 scores were normalized, with the neutral state assigned as the maximum reference point and the p-doped state as the minimum value, as demonstrated in Figure 2d, from which it is also evident that both the normalized band ratios and PC1 are indicative of the doping state. The results reveal that MEH-PPV shows a high doping state at 7 V. However, at higher potentials, we observe a partial dedoping phenomenon, characterized by a slight increase in both the band ratios and PC1. There are two plausible reasons for this dedoping phenomenon. First, it may be attributed to unintentional oxygen and water vapor doping in the active material during device preparation^8,67^ Such unintended doping can facilitate degradation mechanisms in organic devices.^68^ This degradation can be coupled to a photochemical reaction, increasing the possibility of backbone rearrangement and oxidation.^69^ Second, this phenomenon may also result from short-term degradation occurring near the electrodes as a side reaction during operation under applied bias.^9,70^

**Figure 4:**
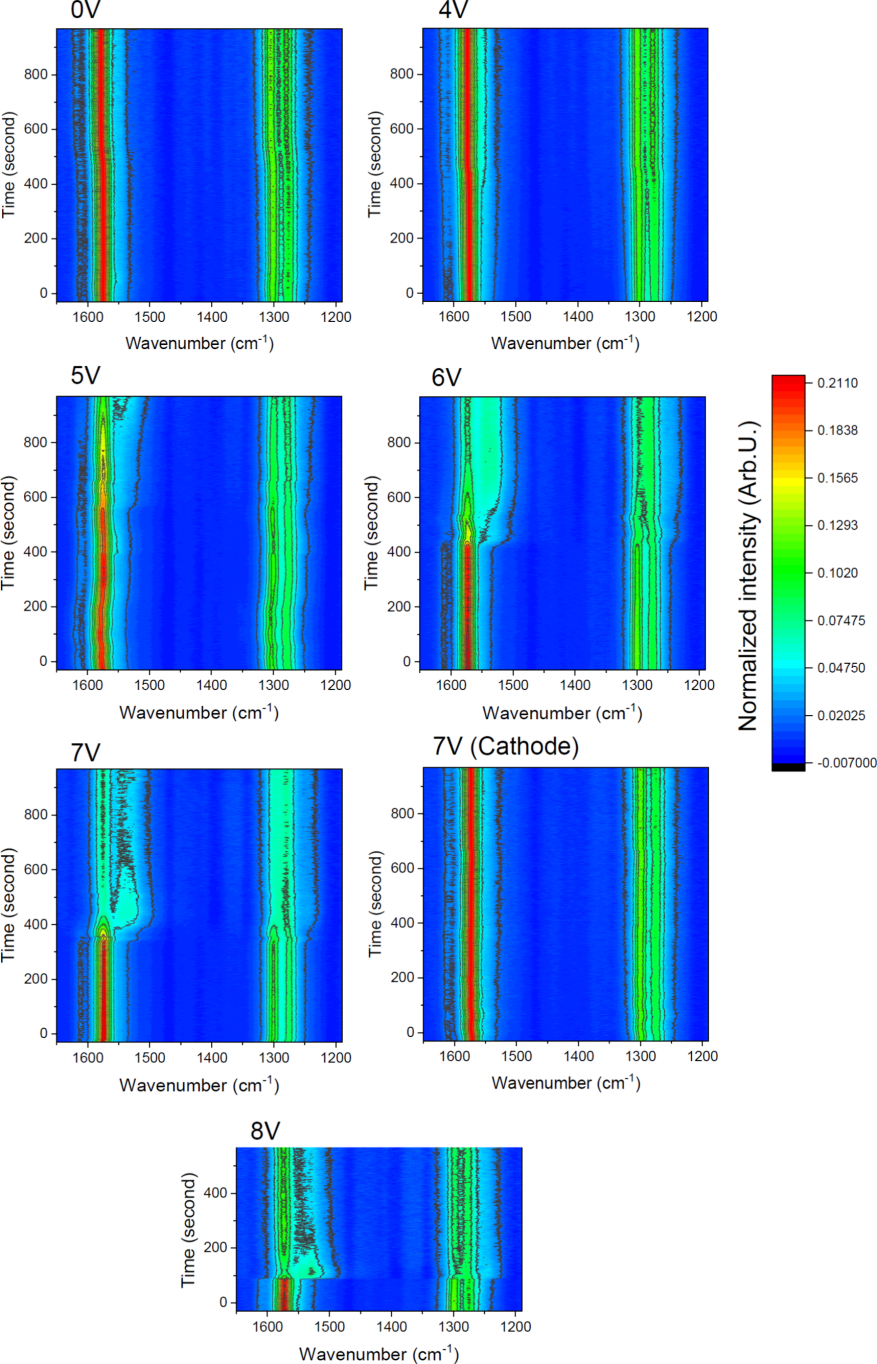
Time maps of the Raman spectra of the active material near the anode (and also the cathode for 7 V) electrodes of the LEC devices under different applied bias (λ_0_ = 785 nm, 10 mW, 1 s).

The conduction mechanism in organic mixed ionic–electronic conductors (OMIECs) can be complex, and the dynamic relationship between ionic and electronic transport is often not well understood. Ions are introduced as free species during device operation, and their transport, particularly diffusion, plays a crucial role in turn-on time, polymer doping, and the formation of p–n junction^1^ in LEC devices. To understand the diffusion of the triflate anion, we can concentrate on the doping of MEH-PPV as an indicator of ion transport within the active layer. We applied different biases while continuously recording Raman spectra near the anode using a 1 s time window. Data was captured at room temperature for 1000 s with the time–current curves shown in Figure S8. Based on the short time window, the obtained data are very noisy and the intensities of the spectra are not the same. Due to this behavior, we smoothed and baseline-corrected the spectra as explained previously. Then, the corrected spectra were vector-normalized in the range 900–1750 cm^–1^ for more accurate evaluation of spectral changes. This approach enabled us to dynamically monitor the doping profiles of MEH-PPV by following benzoid-quinoid ring conversion during p-doping. As previously discussed, the primary peaks attributed to phenyl ring vibrations exhibit shifts from their centered positions at 1580 and 1307 cm^–1^ to lower wavenumbers, approximately 1536 and 1289 cm^–1^, respectively. To further investigate this phenyl ring conversion, we have plotted the Raman spectra against time in the spectral range 1200–1750 cm^–1^, as shown in Figure 4.

Before applying the potential, we obtained spectra of the active material under 0 V for the same duration to detect any degradation caused by the laser. As is evident from Figure 4 (0 V), these spectra did not exhibit any changes. We used this data set as a base reference for further PCA analysis. After that, we applied biases from 4 to 8 V. The results, presented in Figure 4, indicate only weak evidence of structural change of MEH-PPV due to p-doping as a result of anion transfer near the anode when a 4 V bias is applied, which can be seen as a small change after 400 s. When a 5 V bias was applied, a spectral transformation attributed to MEH-PPV ring conversion became noticeable after approximately 550 s. This spectral change was relatively weak and exhibited a gradual increase, continuing through the duration of the experiment. The observed change can be attributed to partial doping effects associated with this voltage. Significantly, the rate of the spectral change (downshift due to benzoid–quinoid ring conversion) increases with the application of higher biases, indicating a more pronounced doping effect under stronger biases. Under a 6 V bias, spectral changes begin around 430 s, which can be associated with the introduction of triflate anions into the anode side and the subsequent doping of MEH-PPV. At this bias level, the spectral map stabilizes after approximately 750 s, suggesting that the polymer has reached a high doping level, likely attributed to the formation of a p–n junction. Under a 7 V bias, the spectral changes due to polymer doping commence around 350 s after the bias is applied and reach a steady state between 650 and 700 s. To compare n- and p-doping, we recorded time-resolved Raman spectra of the active material also near the cathode under a 7 V bias. The results revealed no significant changes upon the application of potential, attributed to the similarity in benzoid ring structure between neutral and reduced MEH-PPV as we have previously reported.^35^ Furthermore, the results did not indicate any spectral changes resulting from material degradation due to electrochemical cathodic side reactions.^9^ Finally, the turn-on time decreases with increasing applied potential, reaching approximately 90 s under an 8 V bias. This decrease in turn-on time is attributed to the accelerated diffusion of triflate ions within the active material in the interelectrode gap.

**Figure 5:**
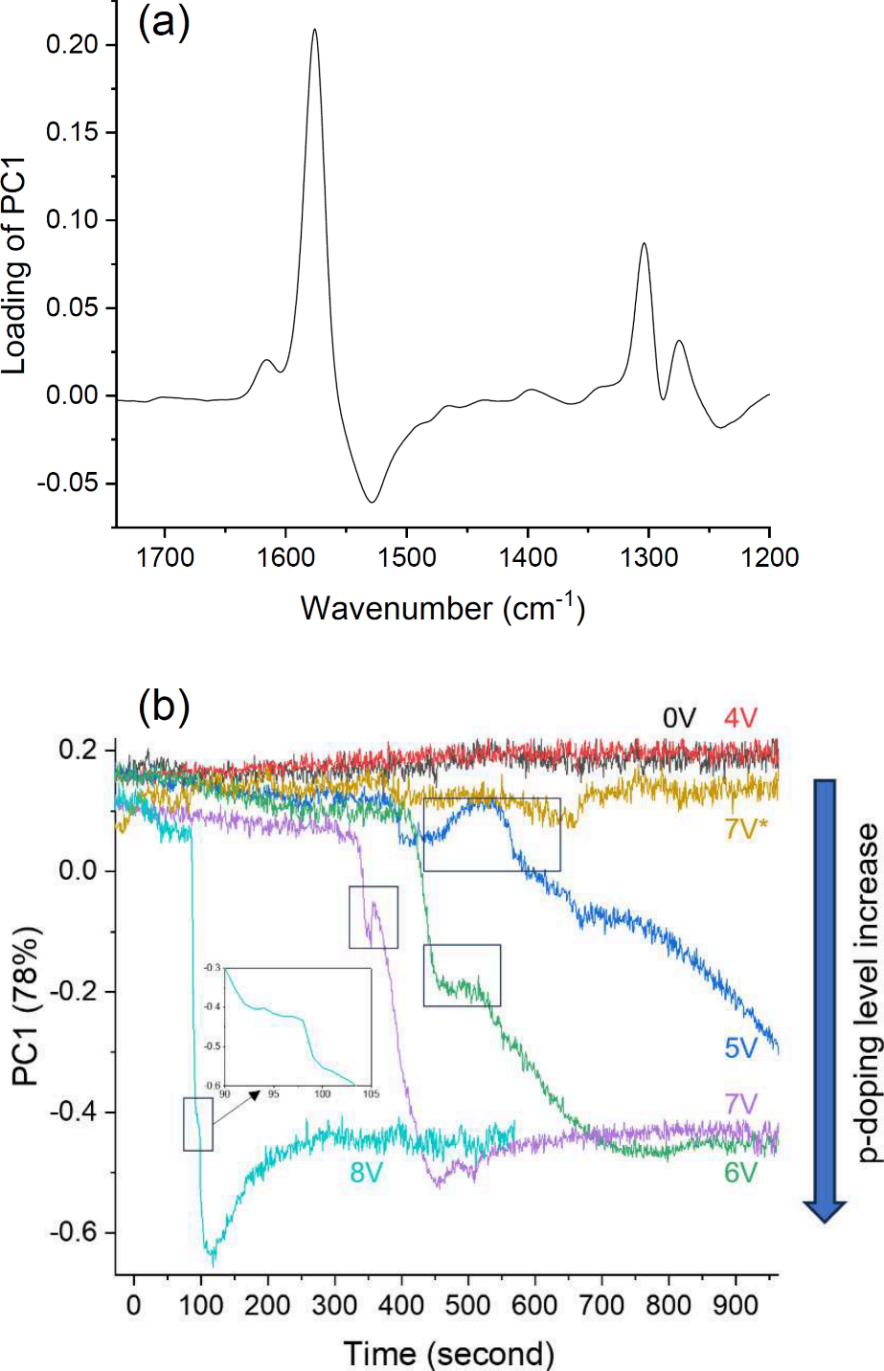
Results of the PCA. (a) Loading plot versus wavenumber and (b) score plot versus operating time for PC1 of the active material near the anode electrodes in the LEC devices under different applied bias, with 7 V* indicating a data series from the cathode side.

To clarify the doping process of MEH-PPV through triflate anion diffusion under applied bias in LEC devices, we conducted a principal component analysis of the data presented in Figure 4 within the spectral range of 1750–1200 cm^–1^. The first principal component (PC1), which captures most of the variance in the Raman data, enables the differentiation of various doping states of MEH-PPV in LEC devices. This differentiation is evident from corresponding data points along the PC1 score, explaining 78% of the variance within the analyzed spectral region. In the loading plot of PC1 (Figure 5a), positive peaks correspond to identical bands arising from the benzoid structure of the phenyl ring, which are indicative of both neutral and reduced (n-doped) MEH-PPV. Conversely, negative peaks in the plot correspond to the quinoid ring formation of MEH-PPV, corresponding to p-doping. As indicated by the PC1 scores in Figure 5b, this analysis reveals that p-doping is initiated partially under a 5 V bias and intensifies with increasing applied bias, leading to a shorter turn-on time. Under an 8 V bias, a high level of doping is observed approximately 90 s after applying the bias, reaching its peak around 120 s after application. Subsequently, the doping level decreases, suggesting a dedoping effect. This dedoping phenomenon may be associated with the degradation of the active material near the anode, as previously discussed. The PC1 curves for biases of 6, 7, and 8 V converge to the same doping level (PC1 value). This level of doping can be regarded as the establishment of a p–n junction and represents a stable operating state for the LEC devices. The PC1 curves corresponding to applied biases ranging from 5 to 8 V exhibit a transient deceleration in the doping process, as indicated within the boxed regions in Figure 5b. This behavior may be linked to the complex conduction mechanism in OMIECs. One plausible explanation for this dedoping phenomenon is the slow electrochemical doping in polymer-ion mixed materials, caused by poor hole transport at low doping levels due to heterogeneous disorder of mixed conducting polymers. This limitation could lead to a slower rate of achieving a steady state in the devices.^2,3^

**Figure 6:**
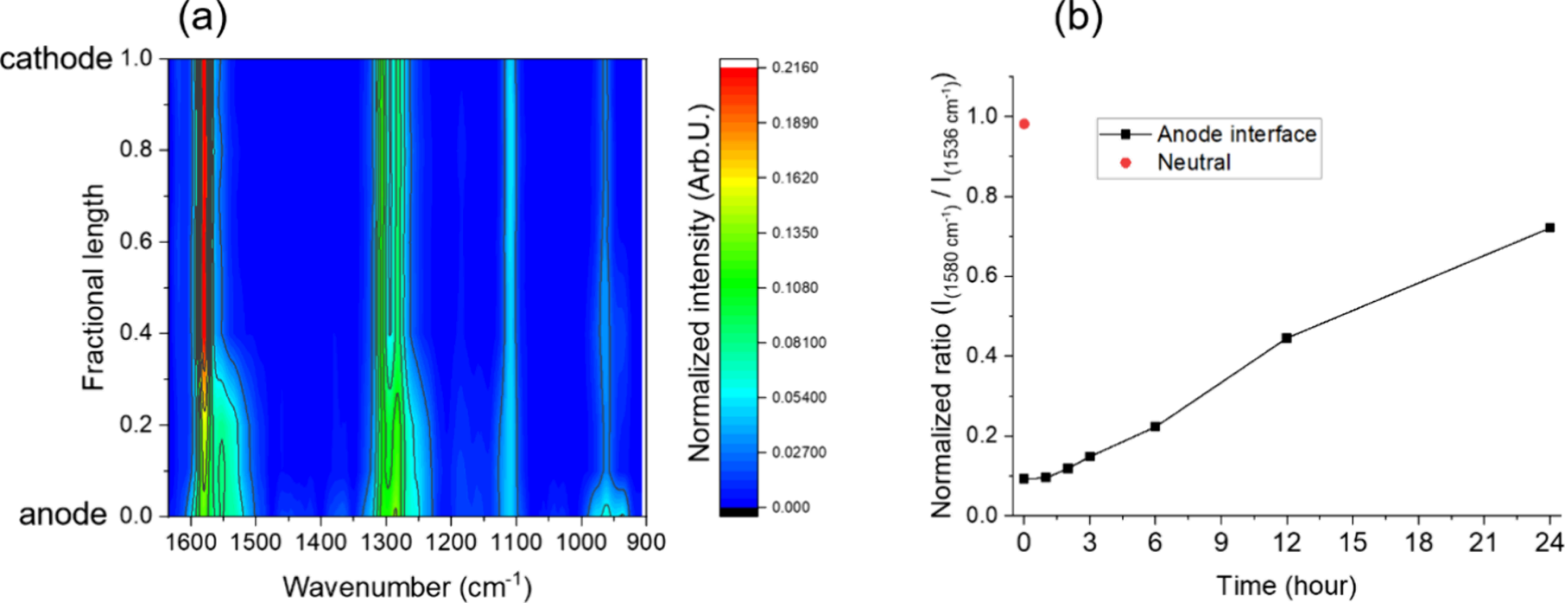
(a) Normalized Raman spectra of the active material for positions along the fractional interelectrode gap right after a 7 V bias was removed, where 0 corresponds to the anode interface and 1 is the cathode interface (λ_0_ = 785 nm, 5 mW, 60 s/spectrum, total acquisition time ca. 15 min). The figure was prepared from spectra obtained at 11 equispaced points between the electrodes. (b) Normalized band intensity ratios of the active material at the anode interface during 24 h after turning off the applied bias and from pristine active material (neutral, red marker).

To obtain a doping profile of MEH-PPV in a fixed-junction LEC device, a 7 V bias was applied to the LEC device at room temperature for 1 h. Subsequently, the bias was turned off, and the LEC device was maintained as an open circuit, while Raman spectra were captured across the interelectrode gap. The normalized Raman spectra are presented in Figure 6a, where 0 corresponds to the anode interface and 1 represents the cathode interface. The results indicate that the active material is p-doped, as evidenced by a wavenumber downshift (consistent with the transformation from benzoid to quinoid phenyl ring structure, as discussed previously) near the anode, extending up to approximately 0.2–0.3 fractional lengths from the anode side. Beyond this point, p-doping drops significantly, marking the formation of a junction between the n-doped and p-doped regions, and the results are in good agreement with the electrochemical doping model. After turning off the device, Raman spectra of the active material at the anode interface, within the p-doped region, were captured during the initial 24 h (see Figure S9). For these spectra and the spectrum of pristine active material, the normalized ratio of the intensity of the neutral phenyl ring (1580 cm^–1^) to that of the p-doped phenyl ring (1536 cm^–1^) was calculated. Figure 6b illustrates that after 24 h, the active material at the anode interface has partially dedoped, although some evidence of p-doping remains. This suggests that the junction is not entirely reversible at room temperature.

## 4 Conclusions

We used surface-enhanced Raman spectroscopy for *in situ* spectroelectrochemical characterization of organic mixed ionic–electronic conductors (OMIECs) by tracking the doping profile in LEC devices. SERS enhancement was achieved via a 40 Å thin thermally evaporated gold film, which spontaneously formed nanoparticles in contact with the substrate, providing an enhancement. This approach enables studies of the doping dynamics in LECs, allowing us to elucidate not only the initial and final stages but also the intermediate steps Raman spectra from regions near the working electrode (mainly the anode) under various applied biases, allowing us to monitor the p-doping profile of MEH-PPV and polaron formation on the anode side and to determine the turn-on time (550 to 90 s, measured as the time needed to reach maximum doping) for applied biases from 5 to 8 V, all conducted at room temperature. Our observations reveal a nonuniform doping rate, particularly pronounced at lower potentials. This disparity could be attributed to limited hole transport during the early stages of doping, exacerbated by lower bias conditions, potentially arising from heterogeneous disorder within the OMIECs. Additionally, our findings illustrate the degradation of MEH-PPV under electrochemical doping conditions in the presence of water and oxygen molecules. Additionally, we conducted Raman mapping within the interelectrode gap between the anode and cathode in a fixed-junction LEC device, confirming the consistency of our results with the electrochemical doping model.

## Supporting Information Available

Raman spectra of active material from different configurations at 532 nm excitation, UV–vis spectra w/o active material layer, Raman and UV–vis spectra of MEH-PPV and active material, Raman spectra of the active material in working device under different applied biases, images showing electroluminescence under applied potential, structures of the doped and undoped active layer materials and the associated spectral changes, *I*–*V* curve of LEC device under a sweeping bias, *I*–*t* curve of LEC device under different applied biases, Raman spectra of a switched-off LEC during the first 24 h, and calculation of enhancement factors (PDF)
