# Supplementary material for: In Situ Surface-Enhanced Raman Spectroscopy on Organic Mixed Ionic-Electronic Conductors: Tracking Dynamic Doping in Light-Emitting Electrochemical Cells
Source: ACS Appl Mater Interfaces. 2024 May 23;16(22):28938–48. doi: 10.1021/acsami.4c00684 (PMC11163397; doi:10.1021/acsami.4c00684)
Supplement: Supplementary file 1 — am4c00684_si_001.pdf [file am4c00684_si_001.pdf]

Supporting information:

In Situ Surface-Enhanced Raman Spectroscopy on Organic Mixed Ionic-Electronic Conductors: Tracking Dynamic Doping in Light-Emitting Electrochemical Cells

Mohammad Javad Jafari[1], Jonas Oshaug Pedersen[1], Samira Barhemat[2], Thomas Ederth[1,*]

[1] *Division of Biophysics and Bioengineering, IFM, Linköping University, 581 83 Linköping, Sweden.*

[2] *Department of Vision Inspection, Mabema AB, 584 22 Linköping, Sweden.*

* Corresponding author: thomas.ederth@liu.se

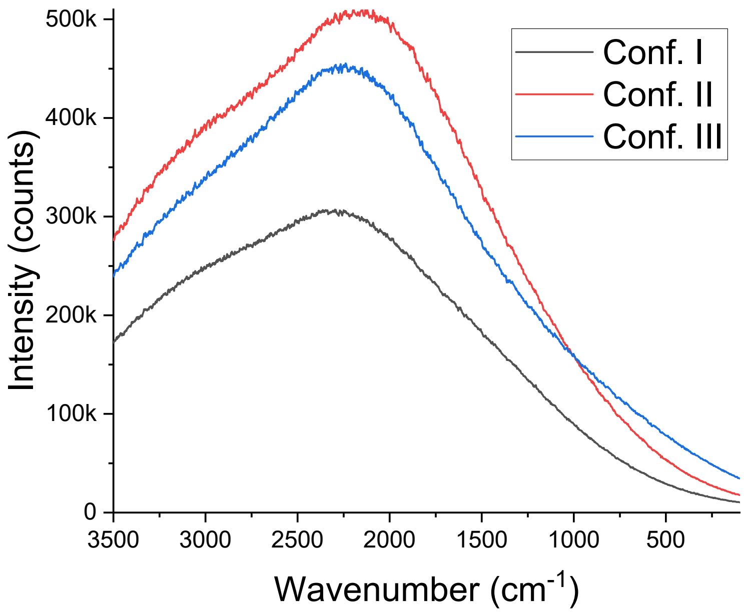

Figure S1. Raman spectra of the active material in different configurations,showing strong fluorescence with the selected excitation light (λ_0 = 532 nm, 0.05 mW, 30 s).

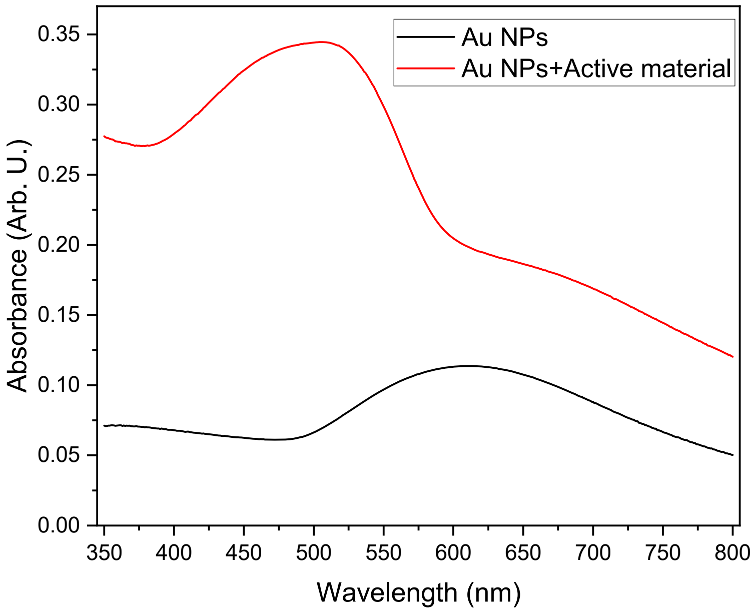

Figure S2. UV/Vis spectra of glass slides with nanoparticles only (black) and nanoparticles + active material (red).

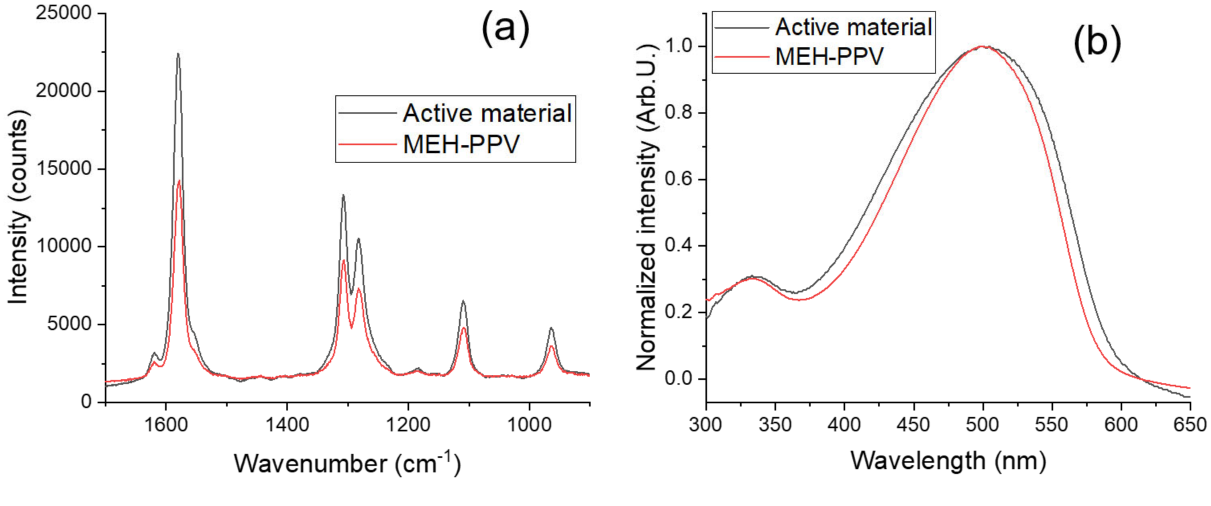

Figure S3. (a) Raman (λ_0 = 785 nm, 10 mW, 60 s) and (b) normalized optical absorption spectra of the active material and MEH-PPV.

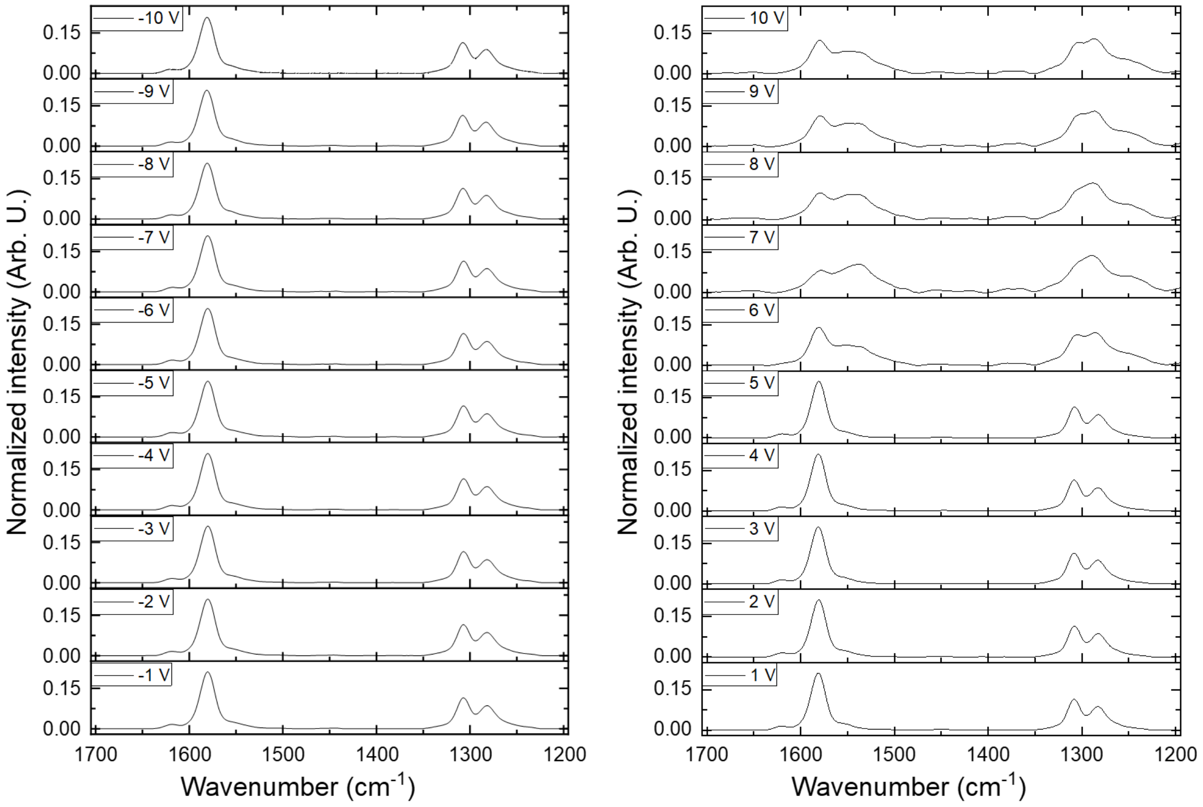

Figure S4. Normalized Raman spectra of the active material near the working electrode under each potential step (λ_0 = 785 nm, 5 mW, 300 s)

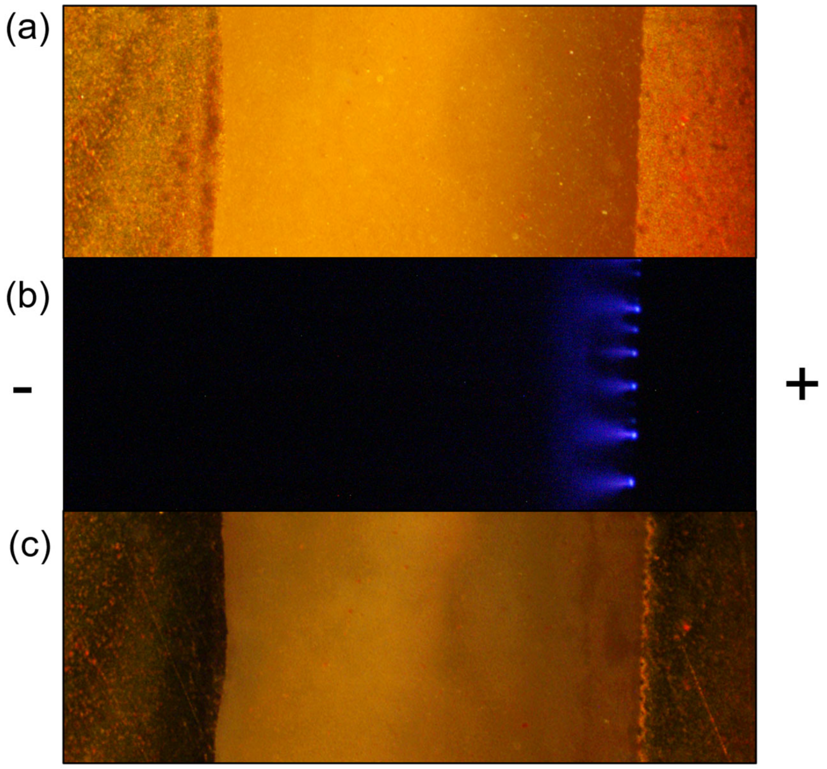

Figure S5. Images of a planar LEC device (a) before applying bias, (b) electroluminescence image of the device under 200V applied potential at room temperature after 1 minute and (c) after applying bias. The thick and inhomogeneous film resulting from the drop casting, as well as the uneven edge of the metal electrode leads to inhomogeneous doping and the formation of an irregularly shaped light-emitting junction, which also changes over time.

On the possible formation of micro-junctions due to gold nanoparticles

The work described in the main text involved applied biases up to 10 V, at this potential no light is emitted, due to the large interelectrode gap. Shorter gaps and higher potentials result in faster formation of a junction, and at the high potentials resulting in emission the rapid formation of the junction prevents detailed studies of the doping dynamics; hence the potentials were kept much lower. At 200 V, light emission appears from a region near the anode (see above), with bright spots from junctions formed in the immediate vicinity of the anode, and a smaller contribution to the luminescence further into the interelectrode area. This smaller, diffuse contribution could be a contribution from microscopic junctions formed between the metal particles used for the SERS enhancement. However, we note that they only fan out from locations where a junction has already been formed. These junctions emerge dynamically, at different positions along the electrode edge, at different points in time, reflecting the heterogeneity of the electrode and the material, presumably due to uneven electrode edges and the uneven film from the drop casting. However, for several reasons, we do not see that these 'microjunctions' should affect the observations in the main text. First, the potentials were significantly lower, and unlikely to suffice for formation of successive junctions. The data in the main text was also acquired at positions within ca 10 μm of the anode edge, and any 'microjunctions' appearing further from the electrode do not affect the data.

A

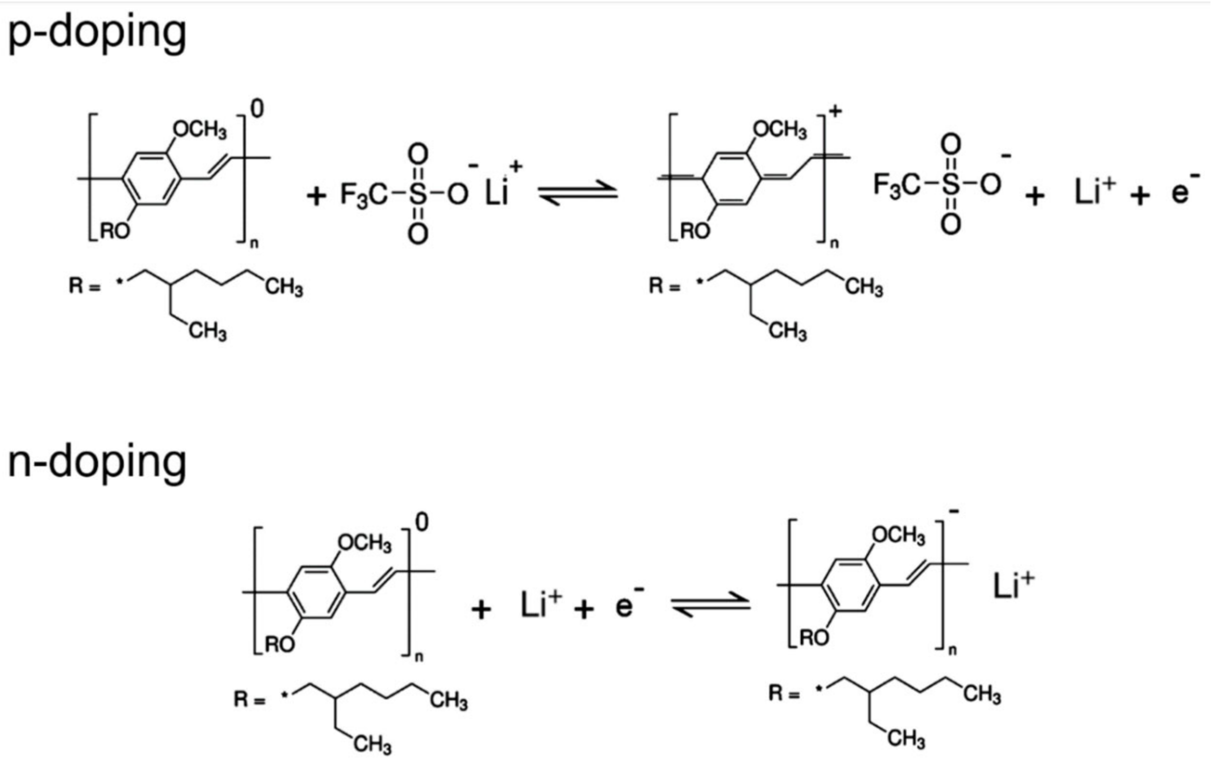

B

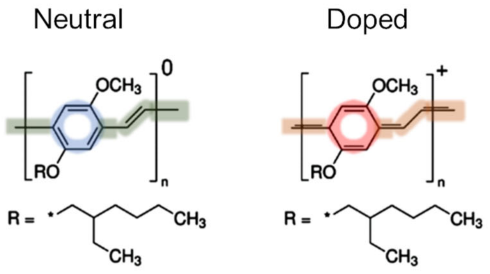

Assignment	Neutral	Doped
Vinylene CC stretch	1621 cm^{-1}	1546 cm^{-1}
Phenyl CC stretch	1580 cm^{-1}	1536 cm^{-1}
Phenyl CC stretch	1307 cm^{-1}	1288 cm^{-1}
Vinylene out-of-plane CH bending	963 cm^{-1}	975 cm^{-1}<br>955 cm^{-1}

Figure S6. A) Doping structural reaction scheme for MEH-PPV. B) Graphical assignment of the spectral changes occurring upon p-doping.

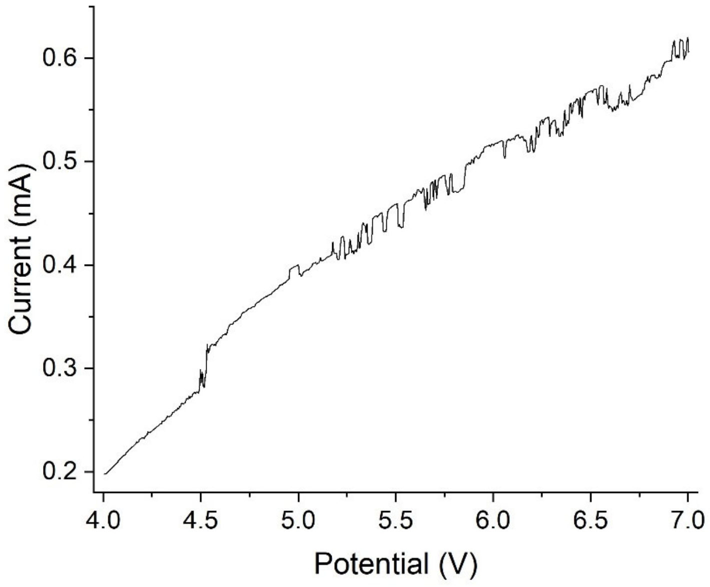

Figure S7. Current-potential curve for the LEC under sweeping bias (1 mV/s).

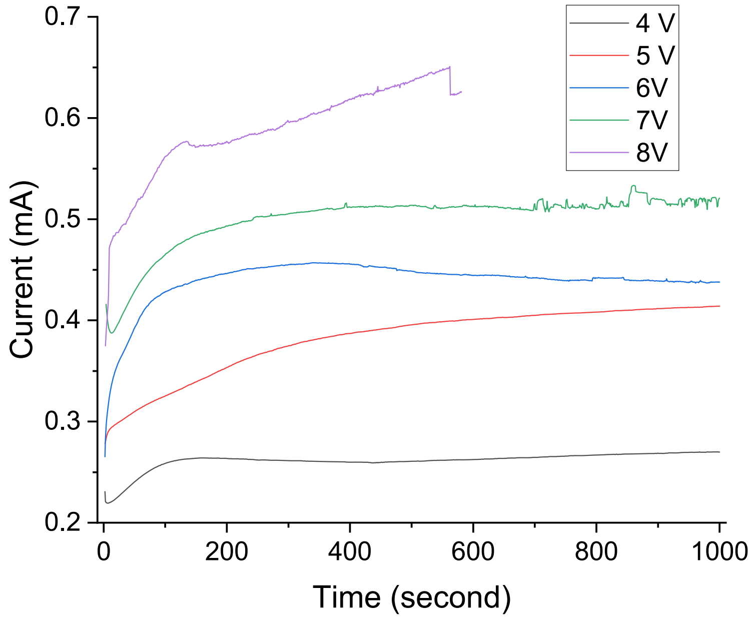

Figure S8. Current versus time for LEC devices under different applied biases.

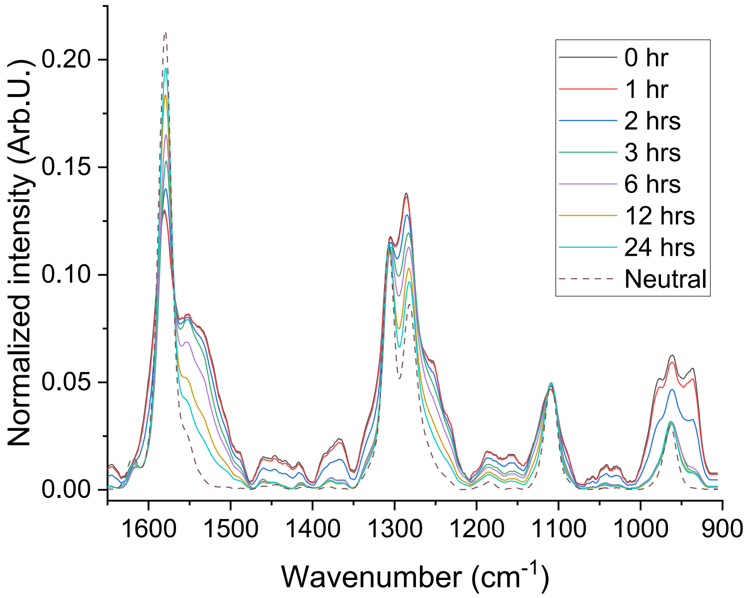

Figure S9. Normalized Raman spectra of the active material from near the anode interface of a frozen-junction device, and a spectrum of the pristine material (Neutral) (λ_0 = 785 nm, 5 mW, 60 s).

Calculation of the enhancement factor

The enhancement factor (EF) was calculated according to the relation

$EF = (I_{SERS}/c_{SERS})/(I_R/c_R)$

where I_{SERS} and I_R are the intensities of the SERS and Raman signals respectively, and c_{SERS} and c_R are the analyte concentrations in the SERS and Raman experiments. I_{SERS}/I_R was taken from the data in Figure 1d in the main article to be a factor 10. The concentrations were replaced with the SERS enhanced volume, V_{SERS}, and laser spot volume, V_R, for SERS and Raman measurements respectively, under the assumption that the amount (or density) of analyte material is comparable in the two experiments. This assumption is reasonable since we see no indications of chemical interaction between the gold and the active material which could lead to different densities close to the NP surface compared to the bulk.

The laser spot volume was calculated as $V_R = A_{laser} \times h$ where A_{laser} is the area of the laser spot and h is the depth of focus. The laser diameter was calculated as $D = 1.22\lambda/NA$, with the numerical aperture NA = 0.7 for the objective used [1]. The resulting spot size is in $A_{laser} = 1.47\ \mu m^2$. h was calculated as $h = 2\lambda/(N.A.)^2 = 3.2\ \mu m$ [2]. Therefore $V_R = 4.704\ \mu m^3$.

The SERS enhanced volume was calculated by considering the nanoparticles as spheres of 100 nm in diameter. The volume outside of the particles that are available for SERS enhancement can then be considered as a spherical shell extending some distance from the particle surface. The distance was set to 5 nm from the NP. This is the maximum distance where SERS enhancement can be determined for spherical gold nanoparticles of this size [3]. Because many of our particles are smaller than 100 nm and therefore have a smaller sensing volume, this provides a conservative estimate of the EF by slightly overestimating the enhancing volume. The shell volume is $(4/3)\pi(R^3-r^3) = 8.253 \times 10^{-5}\ \mu m^3$ where R is the outer radius and r is the inner radius. Under the assumption that approximately 10^4 particles are within the laser spot size (as estimated from analysis of SEM images like Figure 1c using ImageJ), the total volume becomes $V_{SERS} = 0.8253\ \mu m^3$.

Therefore the EF becomes $(I_{SERS}/I_R) \times V_R/V_{SERS} = 10 \times (4.704)/(0.8253) = 57$.

References

1. Xu, Z., et al., Topic Review: Application of Raman Spectroscopy Characterization in Micro/Nano-Machining. Micromachines, 2018. 9(7): p. 361.

2. Tao, C.-a., et al., Cucurbit [n] urils as a SERS hot-spot nanocontainer through bridging gold nanoparticles. Chemical Communications, 2011. 47(35): p. 9867-9869.

3. Kumari, G., J. Kandula, and C. Narayana, How far can we probe by SERS? The Journal of Physical Chemistry C, 2015. 119(34): p. 20057-20064.
